# Stroke severity modified the effect of chronic atrial fibrillation on the outcome of thrombolytic therapy

**DOI:** 10.1097/MD.0000000000029322

**Published:** 2022-06-30

**Authors:** Rui Shao, Zengna Wang, Hongfeng Shi, Yan Li, Yingle Zhuang, Juan Xu, Min Xu

**Affiliations:** Neurological Intensive Care Department, Shengli Oilfield Central Hospital, Dongying City, Shandong Province, China.

**Keywords:** chronic atrial fibrillation, outcome, stroke severity, thrombolysis

## Abstract

There is conflicting information regarding the impact of chronic atrial fibrillation (AF) on the outcomes of thrombolyzed patients with stroke. This study was designed to identify high-risk patients with chronic AF who had undergone thrombolysis treatment and to explore whether the baseline National Institutes of Health Stroke Scale (NIHSS) could be used to distinguish poor clinical outcomes in thrombolyzed patients.

A total of 164 acute ischemic stroke patients with chronic AF were enrolled in this study. The patients were categorized as having poor or favorable outcomes. A favorable 90-day outcome was defined as a modified Rankin Scale (mRS) score ≤2.

Our study showed that the baseline NIHSS score of patients with poor functional recovery (mRS >2) was significantly higher than that of patients with favorable outcomes (median 16 vs 12). Receiver operating characteristic (ROC) curve analysis of mRS score showed that a baseline NIHSS score of 14 was the optimal threshold for predicting unfavorable outcomes in patients with chronic AF. Multivariate logistic regression analysis showed that baseline NIHSS score >14 was independently associated with poor outcomes (odds ratio = 4.182, 95% confidence interval 2.092–8.361).

Our study showed that stroke severity modified the effect of chronic AF on the outcome of thrombolytic therapy. The approach of stratifying stroke severity may be used to evaluate treatment strategies for decision making in intravenous thrombolytic therapy for acute stroke with chronic AF.

## 1. Introduction

Despite reductions in mortality and long-term disability over the last decade, acute ischemic stroke (AIS) remains the third leading cause of death and disability worldwide, causing enormous social and economic consequences.^[1,2]^ AIS has a high incidence, especially in patients with atrial fibrillation (AF).^[3]^ AF is an independent risk predictor for ischemic stroke and raises its incidence nearly 5-fold.^[4]^ Patients with AF who suffer stroke appear to have worse outcomes (more disability and greater mortality) than those who suffer ischemic stroke in the absence of AF.^[5–7]^

Early thrombolysis with intravenous recombinant tissue plasminogen activator (rtPA) can increase the odds of good functional outcomes in AIS patients, and 1 in 3 ischemic stroke patients treated with early thrombolysis achieves a significant benefit.^[8]^ It is still controversial whether acute ischemic stroke patients with AF should receive rtPA therapy, especially patients with chronic AF.^[9–11]^ A study demonstrated that patients with chronic AF had worse stroke outcomes after intravenous thrombolysis than those without AF.^[12]^ However, in this study, subgroup analyses were not performed to identify high-risk patients with chronic AF who were vulnerable to adverse stroke outcomes with rtPA treatment.

We assumed that acute ischemic stroke severity might alter the effect of chronic AF on the outcome of thrombolytic therapy. To date, however, there has been no study exploring the importance of stratification by baseline National Institutes of Health Stroke Scale (NIHSS) score when evaluating the influence of chronic AF on the outcomes of intravenous rtPA therapy. Given the above consideration, this study was designed to identify high-risk thrombolysis recipients with chronic AF and to explore whether baseline NIHSS scores could be used to predict poor clinical outcomes in thrombolyzed patients.

## 2. Methods

Between August 2017 and April 2021, consecutive chronic AF patients with acute anterior circulation ischemic stroke who received rtPA treatment within 3 hours of symptom onset at Shengli Oilfield Central Hospital, an urban university tertiary hospital and national advanced stroke center, were admitted to our study. Patients who underwent endovascular treatment and patients with valvular atrial fibrillation were excluded. Valvular AF refers to patients with mitral stenosis or artificial heart valves. All thrombolyzed patients were treated according to the standard protocol of the AHA/ASA guidelines.^[1]^ Clinical characteristics, including demographic characteristics, past medical history, drug usage, and baseline NIHSS scores, were collected. This study was approved by the Shengli Oilfield Central Hospital Ethics Committee (approval no. Q/ZXYY—ZY—YWB—LL202137). Written informed consents were obtained from the patients. Consents for patients who were unable to consent were provided by first-degree relatives. AF was diagnosed by use of a 12-lead electrocardiogram. Persistent AF that was sustained beyond 3 months was considered chronic.

All thrombolyzed patients underwent computed tomography scans before treatment with rtPA treatment, and the scans were repeated 24 hours later; additional computed tomography (CT) was also performed in case of clinical deterioration. Symptomatic intracranial hemorrhage was defined as any apparent extravascular blood in the brain or within the cranium that was associated with clinical deterioration, as defined by an increase of 4 points or more in the score on the NIHSS.^[2]^ Functional status was assessed 3 months after stroke onset using the modified Rankin Scale (mRS). Patients were classified as having poor or favorable outcomes according to the mRS; a poor outcome was defined as an mRS score >2.

Unadjusted baseline groups were compared using the Pearson *χ*2 test, the Mann–Whitney U test or the 2-sample t test depending on the nature and distribution of the data. Multigroup comparisons were conducted using the Kruskal-Wallis 1-way analysis. Receiver operating characteristic (ROC) curves were conducted, and the predictive qualities of the NIHSS score was evaluated by the area under the ROC curve (AUC). Dichotomized outcome measures were conducted using binary logistic regression.

*P* < 0.05 was considered to be statistically significant. Analyses were undertaken using the statistical software SPSS 19.0 (SPSS Inc., Chicago, IL).

## 3. Results

**Figure 1.**
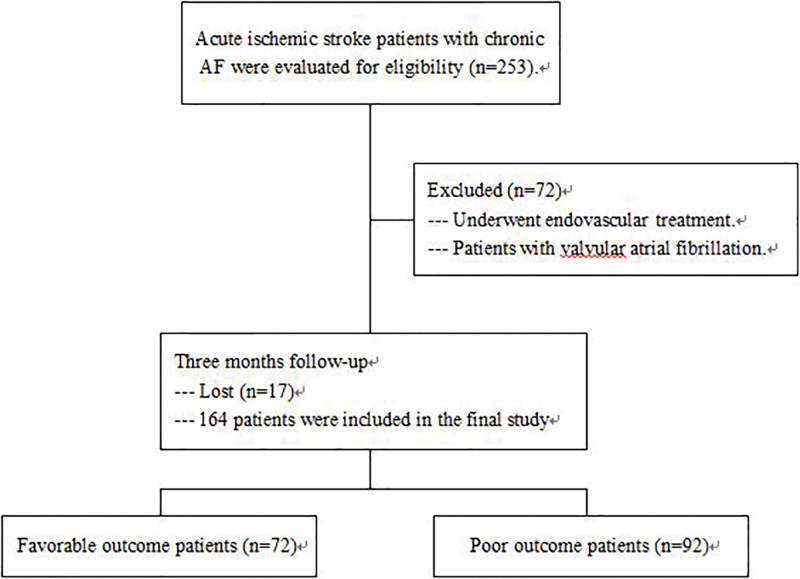
Research flow chart.

A total of 164 patients with chronic AF who received rtPA treatment were included in the study (Fig. 1). According to the follow-up, 72 patients achieved favorable 90-day functional outcomes, and poor 90-day functional recovery was found in 92 patients. Their characteristics are given in Table 1. There were no significant differences in age, sex, or medical history among the patients in the 2 groups. The baseline NIHSS scores of patients with poor functional recovery (mRS > 2) were significantly higher than those of patients with favorable outcomes (median 16 vs 12).

**Table 1:** Comparison between favorable and poor outcome patients with chronic AF treated with rt-PA.

Characteristic	Favorable outcome patients (n = 72)	Poor outcome patients (n = 92)	*P* value
Age (years) median(IQR)	71 (66–78)	68 (62–78)	0.144
Male n(%)	42 (58%)	56 (61%)	0.742
Medical history, n(%)			
Hypertension	17 (24%)	28 (30%)	0.331
Diabetes mellitus	23 (32%)	26 (28%)	0.609
Previous stroke	21 (29%)	19 (21%)	0.208
Smoke	27 (38%)	34 (37%)	0.943
Medications, n(%)			
Anticoagulation use	3 (4%)	7 (8%)	0.558
Antiplatelet use	19 (26%)	25 (27%)	0.910
Glucose(mmol/L) median(IQR)	8.4 (6.7–9.8)	8.1 (6.9–9.6)	0.749
Baseline NIHSS median(IQR)	12 (8–16)	16 (12–21)	0.001
CHADS–VASc score	2 (2–3)	2 (1–3)	0.178
Symptomatic ICH, n(%)	5 (7%)	13 (14%)	0.144

AF = atrial fibrillation, ICH = intracranial hemorrhage, IQR = interquartile range, NIHSS = National Institutes of Health Stroke Scale, rt-PA = recombinant tissue plasminogen activator.

**Figure 2.**
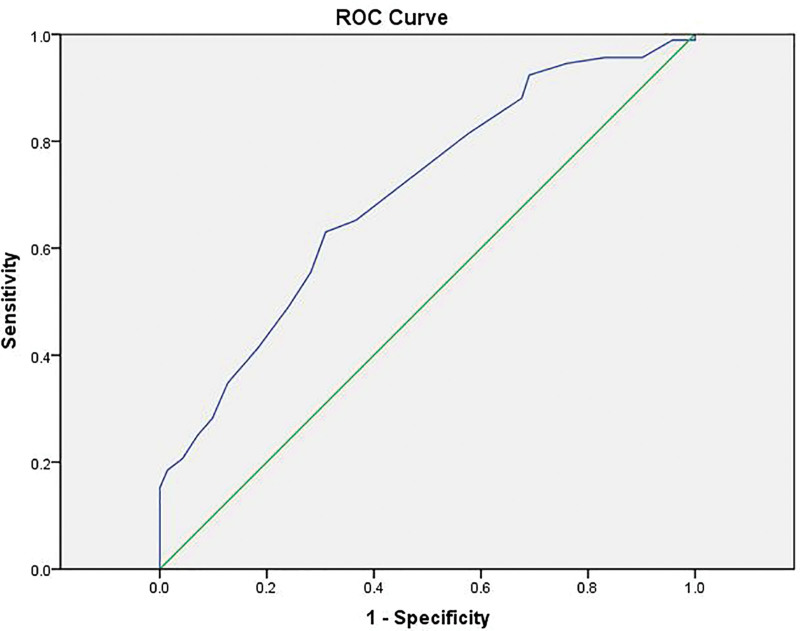
The ROC curve analysis showed that the area under the curve (AUC) of baseline NIHSS scores for predicting unfavorable outcomes at 90 days was 0.708. ROC curve = receiver operating characteristic curve.

We established ROC curves and AUCs to evaluate the predictive qualities of NIHSS scores. The ROC curve analysis showed that the AUC of baseline NIHSS for predicting unfavorable outcome at 90 days was 0.708 (Fig. 2). ROC curve analysis showed that a baseline NIHSS of 14 was the optimal threshold for predicting unfavorable outcomes in patients with chronic AF.

**Figure 3.**
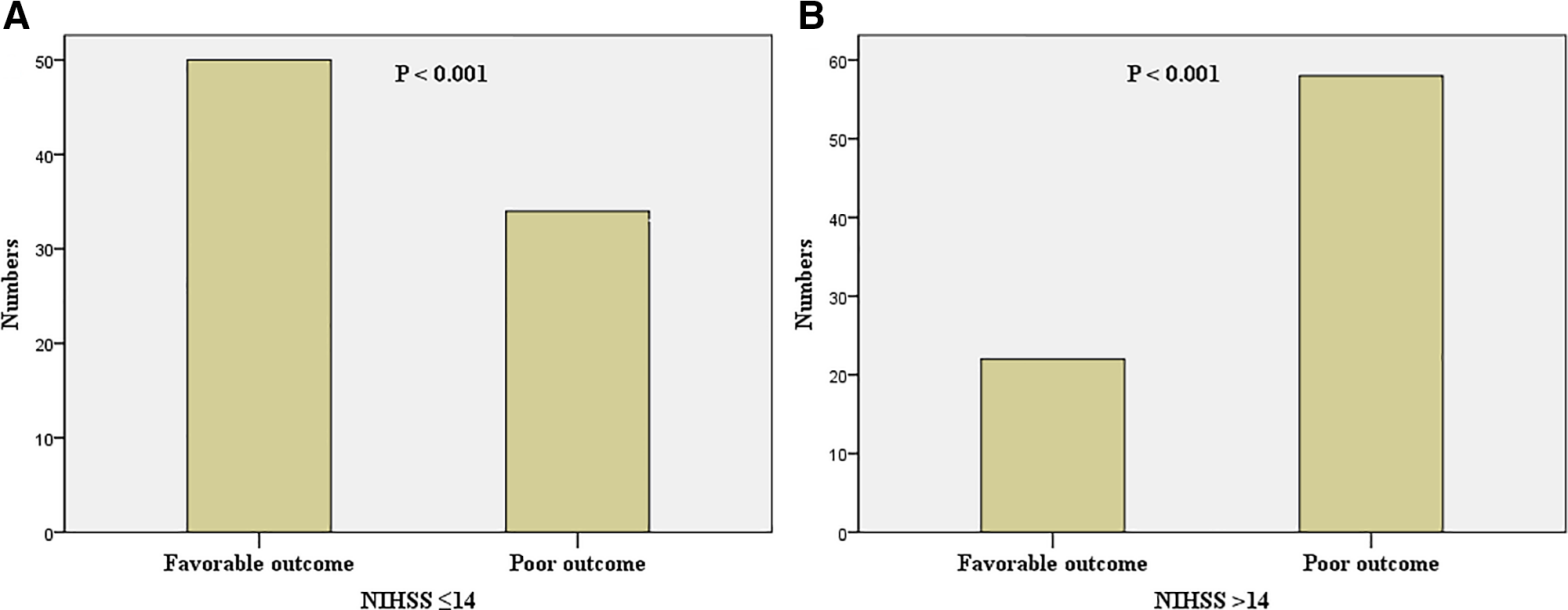
Bar graph showed that there was significantly more favorable prognosis than poor prognoses in patients with an NIHSS score 14 or less, while the outcome was reversed in people with an NIHSS score >14.

**Figure 4.**
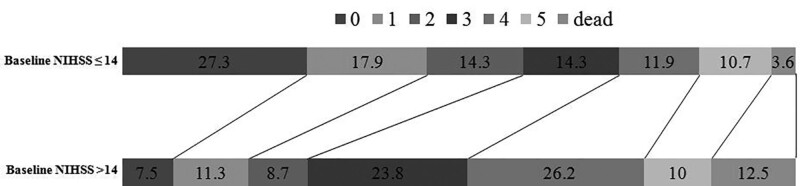
Association of mRS outcome at 90 days with use of alteplase in patients with baseline NIHSS scores ≤14 and those with baseline NIHSS scores of >14. Each box of the horizontal bar corresponds to the mRS category specified by the color code. Numbers in each box denote the percentage of patients having the mRS score corresponding to the box. mRS = modified Rankin Scale, NIHSS = National Institute of Health Stroke Scale.

Using the cutoff value determined by ROC curve analysis, patients with baseline NIHSS scores 14 or less achieved a favorable functional outcome than those with NIHSS scores >14 (59.5% vs 27.5%). Their characteristics are given in Table 2 and Figure 3. In patients with NIHSS scores >14 had a significantly poor functional outcome and a trend toward higher mortality (Table 2). Patients with baseline NIHSS scores 14 or less had a significantly favorable functional 90-day outcome than those with scores >14 (Fig. 4) (*P* < 0.001). When baseline NIHSS score 14 was used as a cutoff for predicting unfavorable outcomes in thrombolyzed patients with AF, it had a sensitivity of 63.0%, a specificity of 69.4%, a positive predictive value (PPV) of 72.5%, and a negative predictive value (NPV) of 59.5%.

**Table 2:** Clinical outcomes of the study patients.

	NIHSS ≤14 (n = 84)	NIHSS >14 (n = 80)	*P* value
Favorable outcome (mRS≤2) at 90 days	50 (59.5%)	22 (27.5%)	< 0.001
Symptomatic ICH	9 (10.7%)	9 (11.3%)	0.913
Mortality	3 (3.6%)	10 (12.5%)	0.068

ICH = intracranial hemorrhage, RS = modified Rankin Scale, NIHSS = National Institutes of Health Stroke Scale.

Univariate and multivariate logistic regression were used to identify baseline NIHSS scores associated with unfavorable outcomes. Multivariate logistic regression analysis showed that baseline NIHSS score of >14 was independently associated with poor outcomes (odds ratio = 4.182, 95%CI 2.092–8.361).

## 4. Discussion

The present study showed that acute ischemic stroke severity altered the effect of chronic AF on the outcome of thrombolytic therapy. We observed that severe stroke patients with chronic AF (baseline NIHSS score >14) may be more prone to develop poor outcomes than those with lower scores. To our knowledge, this is the first study to identify stroke patients with chronic AF according to stroke severity and evaluate the significance of this distinction with respect to outcomes after thrombolytic treatment. Baseline NIHSS thresholds may be used to evaluate treatment strategies for stroke patients with chronic AF.

To promote stroke prevention and control, the Ministry of Health China Stroke Prevention Project Committee (CSPPC) was established in April 2011, which has led to a significant improvement in stroke care.^[13]^ And the prognosis of Chinese stroke patients appears to have improved.^[14]^ The proportion of patient with AF was 6.4% among ischemic stroke patients in China.^[14]^ AF is an independent risk predictor for ischemic stroke and raises its incidence nearly 5-fold.^[4]^ It is important to identify high-risk thrombolysis recipients with chronic AF.

Although international guidelines suggest thrombolysis treatment as a first-line treatment for eligible patients when administered within 4.5 hours after the onset of stroke, only 1 in 3 patients treated by thrombolysis achieves a significant benefit.^[15,16]^ It is still controversial whether acute ischemic stroke patients with chronic AF should receive rtPA therapy. Raymond and colleagues found that patients with chronic AF have worse stroke outcomes than do patients without AF, and the risk of poor outcomes was greater in patients with a longer duration of AF, but those investigators were not able to perform additional subgroup analyses to identify high-risk patients because of the small sample size.^[12]^ Our study found that acute ischemic stroke severity might alter the effect of chronic AF on the outcome of thrombolytic therapy. Severe stroke patients with chronic AF (NIHSS score >14) may be more likely to progress to poor prognosis.

The characteristics of blood clots in patients with chronic AF are still controversial. Stroke patients with chronic AF may have old, large emboli that are resistant to being dissolved with alteplase after reaching intracranial vessels. Our study found that moderate stroke patients treated by thrombolysis may achieve favorable outcomes, but severe stroke patients may not gain any obvious benefit. The reason may be that the emboli of moderate stroke patients split into small fragments after reaching intracranial vessels; these patients would thus achieve significant benefit from alteplase treatment. On the other hand, old, large emboli, which are unlikely to dissolve, may cause a sudden occlusion of large cerebral arteries and may lead to severe stroke with a high NIHSS score. Accordingly, severe stroke patients with AF are resistant to rtPA therapy.

Whether the characteristics of blood clots and their response to rtPA treatment are affected by the chronicity of AF awaits further investigation. Postmortem pathological examination showed that cerebral arteries are mostly occluded by red thrombi in patients with cardioembolic stroke.^[17,18]^ Red thrombi contain some fibrin and erythrocytes which were found more vulnerable to tPA than other thrombi, with a resultant easier to recanalize. However, the chronicity of AF may affect the characteristics of the culprit clots and their resistance to rtPA treatment, and the components of the embolus may be organized and calcified. Several studies have shown that clots associated with AF are more resistant to dissolution with rtPA.^[19,20]^

Our study suggested that stroke severity modified the effect of chronic AF on the outcome of thrombolytic therapy. And severe stroke patients with chronic AF (baseline NIHSS score >14) may be more prone to develop poor outcomes. Studies have demonstrated that patients with a large vessel occlusion in the anterior circulation benefit from endovascular treatment. Further studies are needed to explore whether endovascular treatment alone is better than the bridging strategy of intravenous thrombolysis with alteplase plus endovascular treatment in severe stroke patients with chronic AF.

Our study has several limitations. First, this study was a single-center study, and the current study findings should be confirmed by a multicenter study. Second, the composition of the emboli, which is significant for the identification of pathophysiological changes, was unclear in chronic AF patients. Finally, this study was merely observational, and further studies are required to elaborate on the pathophysiological effects of thrombolysis on thrombi in patients with chronic AF.

## 5. Conclusions

In conclusion, our study showed that stroke severity modified the effect of chronic AF on the outcome of thrombolytic therapy. The approach of stratifying stroke severity may be used to evaluate treatment strategies for decision making for intravenous thrombolysis for acute ischemic stroke with chronic AF.

### Author contributions

Min Xu conceived and designed the study, was involved in drafting the manuscript. Zengna Wang, Hongfeng Shi, Yan Li, Yingle Zhuang, and Juan Xu contributed to collect data and critically revise the manuscript. Rui Shao collected and analyzed the data, performed the statistical analysis, and drafted and critically revised the manuscript. All authors have read and agreed to the published version of the manuscript.

### Acknowledgments

We thank our colleagues in the Neurological Intensive Care Department, Shengli Oilfield Central Hospital for assistance with the study. We sincerely thank all study participants.
